# Graph theory analysis reveals how sickle cell disease impacts neural networks of patients with more severe disease

**DOI:** 10.1016/j.nicl.2018.11.009

**Published:** 2018-11-14

**Authors:** Michelle Case, Sina Shirinpour, Vishal Vijayakumar, Huishi Zhang, Yvonne Datta, Stephen Nelson, Paola Pergami, Deepika S. Darbari, Kalpna Gupta, Bin He

**Affiliations:** a Department of Biomedical Engineering, University of Minnesota, MN, USA; b Department of Electrical and Computer Engineering, University of Minnesota, MN, USA; c Department of Medicine, University of Minnesota, MN, USA; d Department of Hematology Oncology, Children's Hospitals and Clinics of Minnesota, MN, USA; e Department of Neurology, Children's National Health System, Washington, DC, USA; f Division of Hematology, Children's National Health System, Washington, DC, USA; g Department of Biomedical Engineering, Carnegie Mellon University, PA, USA

**Keywords:** Sickle cell disease, Electroencephalography (EEG), Functional magnetic resonance imaging (fMRI), Graph theory, Resting state, SCD, sickle cell disease, VOC, vasoocclusive crisis, fMRI, functional magnetic resonance imaging, EEG, electroencephalography, MNI, Montreal Neurological Institute, FWHM, full width half maximum

## Abstract

Sickle cell disease (SCD) is a hereditary blood disorder associated with many life-threatening comorbidities including cerebral stroke and chronic pain. The long-term effects of this disease may therefore affect the global brain network which is not clearly understood. We performed graph theory analysis of functional networks using non-invasive fMRI and high resolution EEG on thirty-one SCD patients and sixteen healthy controls. Resting state data were analyzed to determine differences between controls and patients with less severe and more severe sickle cell related pain. fMRI results showed that patients with higher pain severity had lower clustering coefficients and local efficiency. The neural network of the more severe patient group behaved like a random network when performing a targeted attack network analysis. EEG results showed the beta1 band had similar results to fMRI resting state data. Our data show that SCD affects the brain on a global level and that graph theory analysis can differentiate between patients with different levels of pain severity.

## Highlights

• Graph theory used to study global impact of long term sickle cell disease on brain.
• EEG and fMRI results compared to study spatial and temporal impact of disease.
• More severe patients have less clustering and efficiency.
• Small world values correlated with past hospitalizations, linking results to pain.

## 1 Introduction

Sickle cell disease (SCD) is a hereditary blood disorder characterized by debilitating acute pain during recurrent and unpredictable episodes of vasoocclusive crisis (VOC) (Ballas et al., 2012; Tran et al., 2017). Additionally, a significant number of patients suffer from chronic pain. Vaso-occlusion caused by the cascade of inflammatory effects induced by sickling of the red blood cells and hemolysis lead to VOC, which can start in infancy and continue throughout life. Other sequelae of SCD include stroke, silent cerebral infarcts, leg ulcers, and acute chest syndrome (Platt et al., 1994; Rees et al., 2010). Chronic pain is common in patients with SCD and is associated with high rates of hospitalization (Aisiku et al., 2009; Ballas et al., 2010; Smith et al., 2008). Sickle pathobiology replete with VOC, global inflammation, hemolysis and impaired vascular biology lead to organ damage and pain (Rees et al., 2010). Therefore, it is likely that brain function is influenced by sickle pathobiology and chronic pain.

Few groups have investigated abnormalities of brain connectivity in SCD patients. Increased functional connectivity to pain processing regions in these patients such as the anterior cingulate cortex, primary somatosensory cortex and periaqueductal gray matter has been reported (Case et al., 2017; Darbari et al., 2015). Altered resting state network connectivity with abnormalities of the default mode network, salience network, and somatosensory network have also been described (Case et al., 2017; Colombatti et al., 2016; Singavi et al., 2016; Sun et al., 2017; Zempsky et al., 2017). These abnormalities correlate with the frequency of hospitalization, considered to be an indicator of disease severity (Case et al., 2017; Darbari et al., 2015). Clinical and animal studies suggest that central sensitization is one of the mechanisms underlying chronic pain in SCD (Campbell et al., 2016; Cataldo et al., 2015). While studies show that SCD alters natural brain dynamics, the overall impact of this disease on global brain connectivity is still not well understood.

Previous studies have mainly focused on specific areas related to pain processing or well-known resting state networks (Baliki et al., 2014; Huishi Zhang et al., 2016; Kucyi and Davis, 2015; Smith et al., 2009). We took the novel approach of analyzing the brain on a global level by means of graph theory. Graph theory analysis involves defining a set of nodes (brain regions) and edges (functional connectivity strength) (He et al., 2011; Bullmore and Sporns, 2009; Wilke et al., 2011). This is a powerful tool that allows unique cortical representations to be assessed, where node selection defines the complexity of the cortical network being examined. Parameters such as clustering coefficient, a measure of local connectivity, characteristic path length, a measure of network efficiency, small world value, a measure that describes the behavior of the graph, along with other parameters have been used to describe brain networks to further understand the mechanisms involved in diseased brain states (Bullmore and Sporns, 2009; Van den Heuvel and Hulshoff Pol, 2010). For example, when migraine, a chronic pain condition, was studied using graph theory analysis, network abnormalities were identified including higher clustering coefficient values and decreased small world values, showing that long-term migraine causes irregular network construction (Liu et al., 2013). Additionally, graph theory showed that female migraine patients tend to have a more dysfunctional network compared to male patients (Liu et al., 2011). Pain has also been shown to have large scale network impacts in other chronic pain conditions (Balenzuela et al., 2010; Mayer et al., 2015).

Therefore, we examined the effect of chronic pain and severity of SCD on brain networks using graph theory. This study was entirely data driven: regions included in a standard brain atlas were included in order to study the global brain network. Imaging was performed using both functional magnetic resonance imaging (fMRI) and electroencephalography (EEG) in order to provide high spatial and temporal resolution of resting state data. We hypothesize that graph theory would detect differences in network connectivity between healthy controls and patients, and differences among patients with less and more severe disease. Prospectively, we investigated how chronic pain can influence network connectivity. We hypothesize that greater pain intensity would cause more dysfunction in the brain network causing less organization and reduced efficiency in the network.

## 2 Methods

### 2.1 Patients

This study was registered as “Functional Neuroimaging of Pain Using EEG and fMRI” at clinicaltrials.gov (registration number was NCT02212691). The patient group includes thirty-one SCD patients recruited across two sites including the University of Minnesota (Minneapolis, MN) and Children's National (Washington DC). Approvals from the Institutional Review Boards at the University of Minnesota and at Children's National were obtained prior to the start of the study. All patients were recruited by hematologists and gave written informed consent before participating in the study. Participants under the age of eighteen gave assent and their parent or legal guardian gave written informed consent. Written informed consent was also obtained from participants to have the research staff access medical history to obtain clinical parameters relating to the patient's general health and disease severity history, where a summary of these parameters is displayed in Table 1 and medications taken by the patients is shown in Table 2.

**Table 1:** Summary of clinical parameters of sickle cell disease patients.⁎

Patient Characteristics	P	Less Severe Group (n = 15)	More Severe Group (n = 16)
Age, years	0.89	21.4 (±5.7)	21.7 (±6.3)
Females, n (%)	0.60	8 (53)	7 (44)
Type HbSS, n (%)	0.49	12 (80)	11 (69)
Type HbSC, n (%)	0.94	2 (13)	2 (12)
Type HbSβ0, n (%)	0.08	0 (0)	3 (19)
Type HbSβ+, n (%)	0.33	1 (7)	0 (0)
Pain score	0.02	0.1 (±0.4)	1.6 (±2.2)
Systolic blood pressure, mmHg	0.43	116.5 (±11.6)	120.1 (±13.1)
Diastolic blood pressure, mmHg	0.49	67.4 (±7.0)	69.4 (±8.7)
Hemoglobin, g/dL	0.41	9.7 (±1.5)	9.3 (±1.6)
Fetal hemoglobin, %	0.08	10.1 (±8.1)	4.7 (±4.8)
Automated absolute reticulocyte count, K/uL	0.16	241.0 (±129.6)	323.9 (±188.9)
White blood cell count, K/uL	0.84	7.6 (±4.6)	7.9 (±4.1)
Platelet count, K/uL	0.67	354.5 (±171.8)	377.6 (±114.8)
Emergency room visits in past 2 years	0.005	1.4 (±1.4)	20.8 (±23.3)
Hospitalizations in past 2 years	<0.001	1.3 (±1.6)	10.9 (±5.9)
Hydroxyurea, n (%)	0.57	10 (67)	9 (56)

⁎ Note: Values are mean ± SD unless otherwise indicated.

**Table 2:** Group summary of medications used by sickle cell patients.

Medications used by multiple patients		Medications used by one patient	
Medication	N	Amlodipine	Naproxen
Acetaminophen	3	Budesonide	Oxymorphone
Albuterol	7	Benztropine	Pseudoephedrine
Aspirin	2	Bisacodyl	Ranitidine
Deferasirox	3	Celebrex + Celecoxib	Rivaroxavan
Diphenhydramine	2	Cholecalciferol	Senna
Docusate	4	Clozapine	Sildenafil
Fluticasone	4	Desyrel + Trazodone	Topiramate
Folate	15	Escitalopram	Ursodiol
Gabapentin	6	Etonogestrel Implant	Zolpidem
Hydromorphone	2	Fondaparinux	
Hydroxyurea	19	Haloperidol	
Ibuprofen	10	Hydroxyzine	
Lisinopril	2	Lansoprazole	
Meloxicam	2	Levalbuterol	
Montelukast	2	Levonorgestrel	
Morphine extended release	2	Loratadine	
Oxycodone	13	Methadone	
Polyethylene glycol	6	Morphine	
Tramadol	2	Mometasone	

Thirty-one patients participated in this study (15 female, 16 male, average age 21.5 ± 5.9 years). Twenty-one patients were recruited at the University of Minnesota and 10 patients were recruited from the Children's National. Patients were separated into two groups based on their medical history. The sum of the number of hospitalizations and the number of emergency department visits in the past two years was used to group patients as less severe or more severe related sickle pain. Patients with less than eight total visits were placed in the less severe group (*n* = 15, 8 female, 7 male, average age 21.4 ± 5.7 years), and patients with eight or more visits were in the more severe group (*n* = 16, 7 female, 9 male, average age 21.7 ± 6.3 years). Eight visits were selected because it was the median value of the patient group. This allowed us to split the patients evenly among the two groups. Resting state fMRI data was recorded for all patients. However, due to impedance issues with EEG, two patients were removed from the resting state EEG analysis. As a result the group parameters for the total patient group (*n* = 29, 14 female, 15 male, average age 21.7 ± 6.1 years), less severe group (*n* = 14, 7 female, 7 male, average age 21.6 ± 5.8 years), and more severe group (*n* = 15, 7 female, 8 male, average age 21.8 ± 6.5 years) changed slightly.

### 2.2 Controls

The control group consisted of sixteen healthy controls (8 female, 8 male, average age 24.6 ± 4.8 years) who voluntarily participated in the study. The ethnicity of the control group was diverse with 6 African Americans, 6 Caucasians, 3 Asians, and 1 Hispanic. The healthy controls were all screened prior to participation to ensure none had neurological diseases, psychiatric diseases, or chronic pain conditions. Written informed consent was obtained from each control prior to participation in the study. Resting state fMRI was recorded from all healthy controls; however, one control was removed from the EEG resting state analysis due to noise. Fifteen subjects (8 female, 7 male, average age 25 ± 4.4 years) had EEG data and were included in the analysis. The control group did not participate in the thermal pain part of this study.

### 2.3 Resting state fMRI and EEG acquisition

**Fig. 1:**
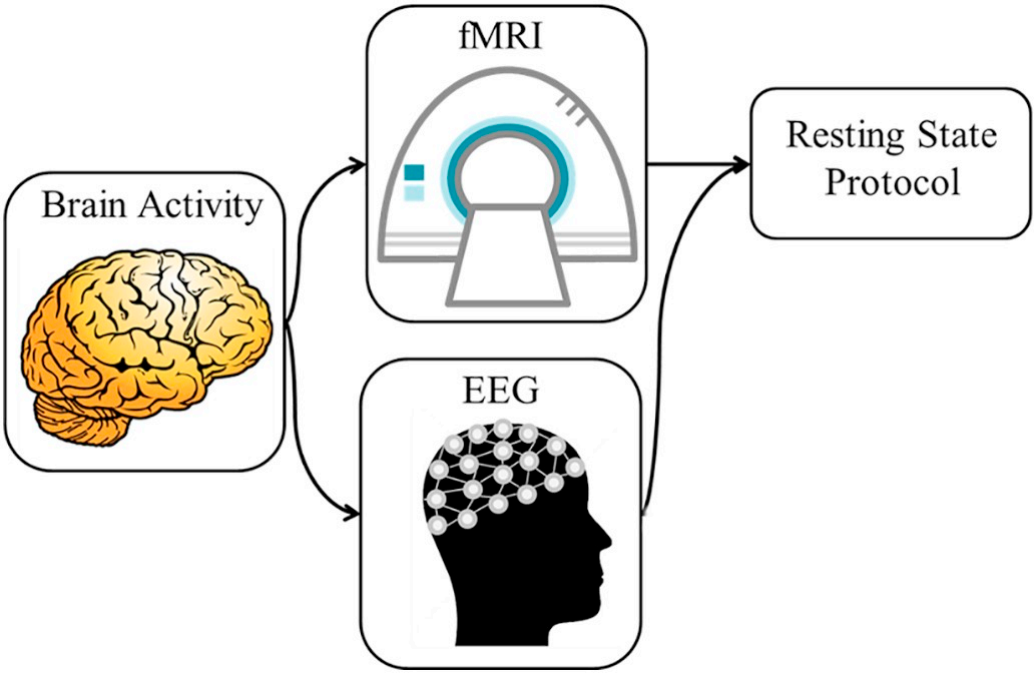
Schematic diagram of experimental procedure. The study involved obtaining resting state data from patients and controls. Both functional magnetic resonance imaging (fMRI) and electroencephalography (EEG) were used to record resting state data. Subjects were asked to let their mind naturally during resting state recordings.

All of the participants in this study completed resting state fMRI acquisition and EEG recording (study schematics in Fig. 1). fMRI data was collected using a, supine position in a 3 T MRI scanner; EEG recordings were performed in a sitting on a chair in a private room. Participants were asked to keep their eyes open while letting their mind wander naturally. Resting state recordings lasted approximately 8 min for fMRI and 10 min for EEG data collection. Participants were asked on the day of the recordings prior to going into the fMRI scanner to verbally rate their pain on a scale from 0 to 10, where 0 indicated no pain and 10 indicated the worst pain imaginable.

### 2.4 fMRI recording and preprocessing

For experiments conducted at the University of Minnesota, a 3 T Siemens Magnetom Trio scanner (Erlangen, Germany) with a sixteen channel head coil was used to record fMRI data on fourteen subjects. The remaining seven patients recruited at the University of Minnesota used a 3 T Siemens Magnetom Prisma scanner (Erlangen, Germany) with a twenty channel head coil for fMRI recordings because of upgrades done at the Center for Magnetic Resonance Research. Anatomical MRI data were obtained for each patient, using a high-resolution T1 sequence with 240 contiguous sagittal slices (slice thickness: 1 mm; matrix size: 256*256; FOV: 256 mm*256 mm; TR/TE = 20 ms/3.3 ms). For functional scans, whole-brain images with blood-oxygen-level-dependent contrast were attained using a gradient echo planar imaging sequence (40 sequential axial slices; slice thickness: 3 mm; TR/TE: 2500 ms/30 ms; flip angle: 90°; matrix size: 64*64; FOV: 192 mm*192 mm). No MR angiography or T2 imaging was performed in this study.

For experiments conducted at Children's National, a 3 T GE Discovery MR750 scanner (Milwaukee, WI) with a thirty-two channel head coil was used to obtain fMRI data from ten patients. Anatomical MRI data was obtained using a high-resolution T1 sequence with 360 contiguous sagittal slices (slice thickness: 1 mm; matrix size: 256*256; FOV: 256 mm*256 mm; TR/TE: 7.2 ms/2.7 ms). For functional scans, whole-brain images with blood-oxygen-level-dependent contrast were attained using a gradient echo planar imaging sequence (52 sequential axial slices; slice thickness: 3 mm; TR/TE: 2000 ms/22 ms; flip angle: 90°; matrix size: 64*64).

The preprocessing steps used in this study have been previously described (Case et al., 2017). Essentially, all fMRI preprocessing was done using SPM12 software (Ashburner et al., 2014), which included slice scan time correction, 3-D motion correction, and temporal filtering. The images were aligned to the anterior-posterior line and normalized into MNI (Montreal Neurological Institute) space. The anatomical and functional images were co-registered and smoothing was performed to three times the size of the original voxels using full width and half maximum (FWHM). The first ten images of the fMRI data were removed so that a steady state of excitation had been reached in the data analyzed.

### 2.5 EEG recording and preprocessing

For resting state experiments conducted at the University of Minnesota, a 64 channel MR-compatible BrainProducts EEG system was used (Gilching, Germany). For resting state experiments conducted at Children's National, a 64 channel Neuroscan system was used (Charlotte, NC). For thermal pain experiments, a 128 channel BioSemi Active-II EEG system was used (Amsterdam, Netherlands). All electrode impedances were below 20 kΩ and all EEG data was recorded at 1 kHz.

The EEG data was processed using the EEGLAB toolbox (Delorme and Makeig, 2004). Visual artifact removal was performed be removing noisy sections of the EEG recordings. Additionally, independent component analysis was used to remove noisy components. The EEG data were resampled to 256 Hz, bandpass filtered between one and 50 Hz, and average referenced. Finally, an automatic artifact rejection toolbox in EEGLAB was used to further clean the data (Gomez-Herrero et al., 2006). The first and last minute of EEG recordings were removed from the resting state data. At least 5 min of artifact-free EEG data was required for participants to be included for further analysis.

### 2.6 Resting state fMRI graph theory analysis

**Fig. 2:**
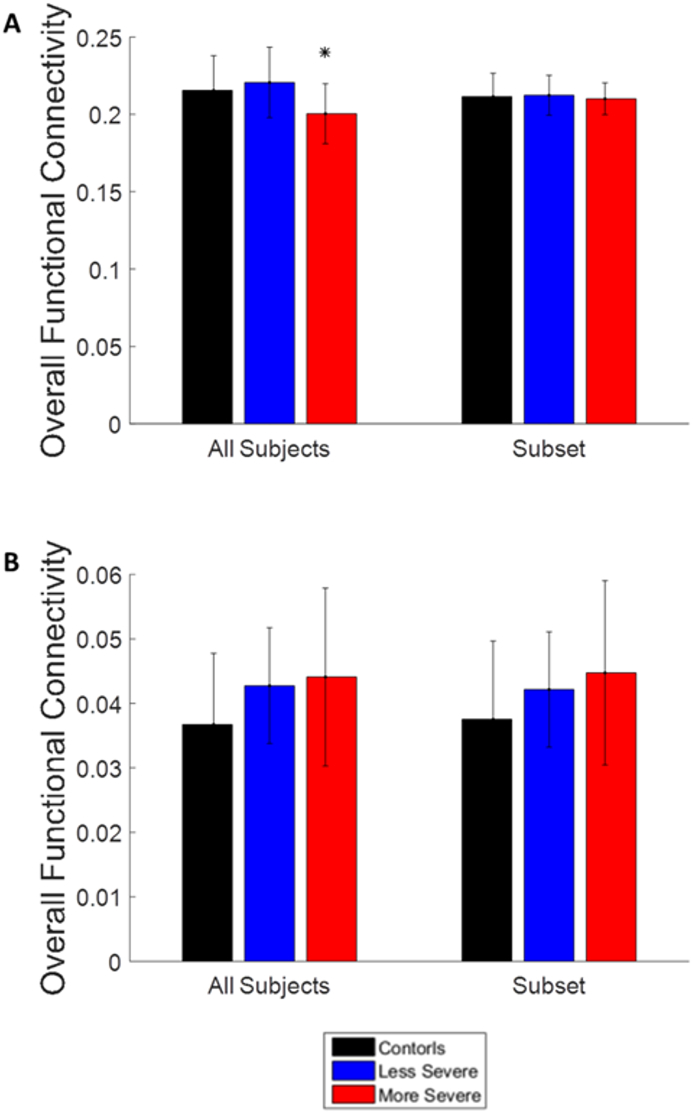
Bar graphs showing overall functional connectivity of subject groups. A. The overall functional connectivity for the fMRI analysis. B. The overall functional connectivity for the EEG analysis. The bars show the average value of the functional connectivity and the error bars show the standard deviation. The * indicates a *p* < .05. No significant differences were observed in the subset groups.

The preprocessed anatomical and functional MRI data was imported into the CONN functional connectivity toolbox (Whitfield-Gabrieli and Nieto-Castanon, 2012). A connectivity matrix was generated for each subject by calculating the correlation coefficient between all of the 136 regions of interest included in the CONN toolbox's Talairach Daemon atlas. Adjacency matrices were generated from each connectivity matrix over a range of sparsity thresholds between 0.10 and 0.30 with a 0.01 increment. Sparsity is defined as the total number of edges in a network divided by the total number of possible edges (Achard and Bullmore, 2007; Liu et al., 2013). Sparsity thresholds ensure the same number of edges is in the graph for each subject. Each adjacency matrix created a graph *G* = (*N*, *E*), where N represents the nodes or brain regions and E represents the edges or functional connections between brain regions. To eliminate potential bias, the overall functional connectivity of each subject was found. There was a significant difference between the less severe and more severe groups, and so a subset group was created that matched functional connectivity across groups (Van den Heuvel et al., 2017). The subset groups did not show any significant differences and each group contained 12 subjects (Fig. 2A). The same subjects were used for the EEG analysis, and no significant differences were found between overall functional connectivity in either the original groups or the subset groups (Fig. 2B). The control and less severe groups contained 11 subjects and the more severe group contained 12 subjects.

Several parameters were calculated from the graph including node degree, clustering coefficient, characteristic path length, small world value, global efficiency, and local efficiency (Bullmore and Sporns, 2009; Menon, 2013; Sporns, 2013). The node degree is the total number of connections at a node (Bullmore and Sporns, 2009; Van den Heuvel and Hulshoff Pol, 2010; Menon, 2013; Sporns, 2013). The clustering coefficient represents the probability that neighbors of a node are also connected to each other (Van den Heuvel and Hulshoff Pol, 2010; Liu et al., 2013; Watts and Strogatz, 1998). The overall clustering coefficient of the network is the average of clustering coefficients across all nodes. The characteristic path length is the average of the shortest path lengths between any pair of nodes in the network (Van den Heuvel and Hulshoff Pol, 2010; Liu et al., 2013; Menon, 2013; Sporns, 2013; Watts and Strogatz, 1998).

The clustering coefficient and characteristic path length were found for a random network consisting of the same number of nodes and edges. These parameters were used to calculate the small world value of the network. A small world network is characterized by high clustering coefficient and short path lengths (Bullmore and Sporns, 2009; Van den Heuvel and Hulshoff Pol, 2010; Menon, 2013). If the small world value is greater than one, the network is considered a small world network (Liu et al., 2011). The global efficiency is the ability of the network to transmit information at a global level (Wang et al., 2010). The local efficiency is the ability of the network to transmit information at a local level and is related to the clustering coefficient (Bullmore and Sporns, 2009; Menon, 2013; Wang et al., 2010). Each of the six parameters was calculated for every subject, and the mean values and standard deviations for each group including controls, less severe, and more severe are reported.

**Fig. 3:**
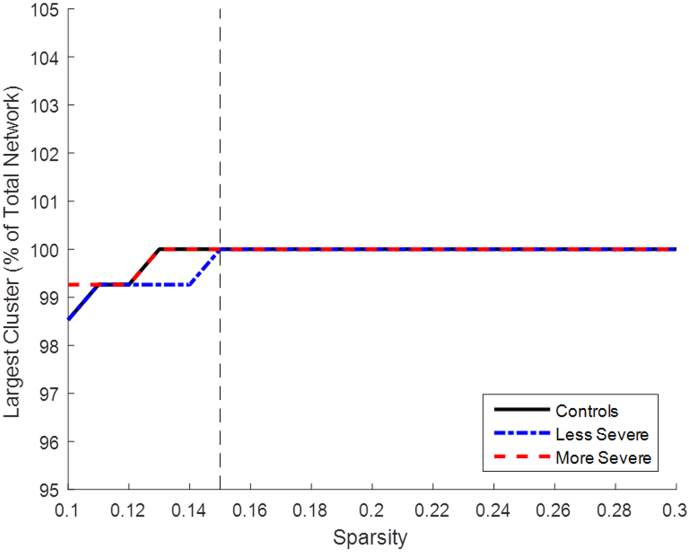
Chart for selecting sparsity level for analysis for fMRI analysis. The chart shows how sparsity level effected how many nodes were included in the network. The smallest sparsity value with all the groups having the complete network connected was chosen for analysis. The sparsity value selected was S = 0.15.

In order to conduct further analysis on the graphs, a fixed sparsity level needed to be selected. The lowest sparsity value where the mean of each group had all nodes connected to the network was selected (Liu et al., 2012), shown in Fig. 3. This allowed each group to have whole network connectivity without the graph being too densely populated. The sparsity value S = 0.15 was selected and is used in all following analysis descriptions.

A robustness analysis was conducted to test the resilience of the networks by using a targeted attack approach described previously (Albert and Barabási, 2002; Liu et al., 2011). The targeted attack involved removing the node with the highest node degree. After the node was removed the size of the largest remaining cluster was found. This process was repeated until all nodes were removed. We found the average response from each group; in addition, we also calculated the response of a random network to the targeted attack procedure.

The six parameters calculated for each network were tested against clinical parameters including the number of emergency department visits and the number of hospitalizations. These parameters were chosen because they have previously been shown to reflect chronic pain severity in sickle cell patients because these values tend to reflect the frequency of acute pain crises (Darbari et al., 2015; Darbari et al., 2012). A linear model was generated by assuming a linear relationship between the graph parameters and the clinical parameters. Only correlations that were determined to be significant are reported.

Finally, the T1 anatomical images were used to assess the gray matter and white matter volume by counting the number of gray matter and white matter voxels. This was done for each group and for each subset group. The anatomical image was segmented using the voxel-based morphometry toolbox, which is an extension of the statistical parametric mapping tool implemented in MATLAB (Ashburner et al., 2014; Ashburner and Friston, 2000).

### 2.7 EEG graph theory analysis

The preprocessed EEG data were imported into the FieldTrip toolbox (Oostenveld et al., 2011). The MRI of Colin27, a realistic standard head model (*Enhancement of MR Images Using Registration for Signal Avera*, n.d.), was used to create a three layer boundary element model (Hamalainen and Sarvas, 1989; He et al., 1987). A three-dimensional source grid with 10 mm resolution was defined from Colin27. The EEG was split into 2 s epochs with 50% overlap. The cross spectrum was calculated using the Fourier Transform and binned into different frequency bands including delta (2–4 Hz), theta (4–8 Hz), alpha (8–12 Hz), beta1 (12–16 Hz), beta2 (16–30 Hz), and gamma (30–50 Hz). The resulting cross-spectral matrix was fed into a beamformer algorithm (Frei et al., 2001; Gross et al., 2001; Veen et al., 1997) for each epoch. A connectivity matrix was calculated from the imaginary part of the coherence spectrum (Nolte et al., 2004). An AAL atlas available from the FieldTrip toolbox with 90 regions of interest was used to parcellate the connectivity matrix. Adjacency matrices were generated from the connectivity matrix for each frequency band for every subject.

**Fig. 4:**
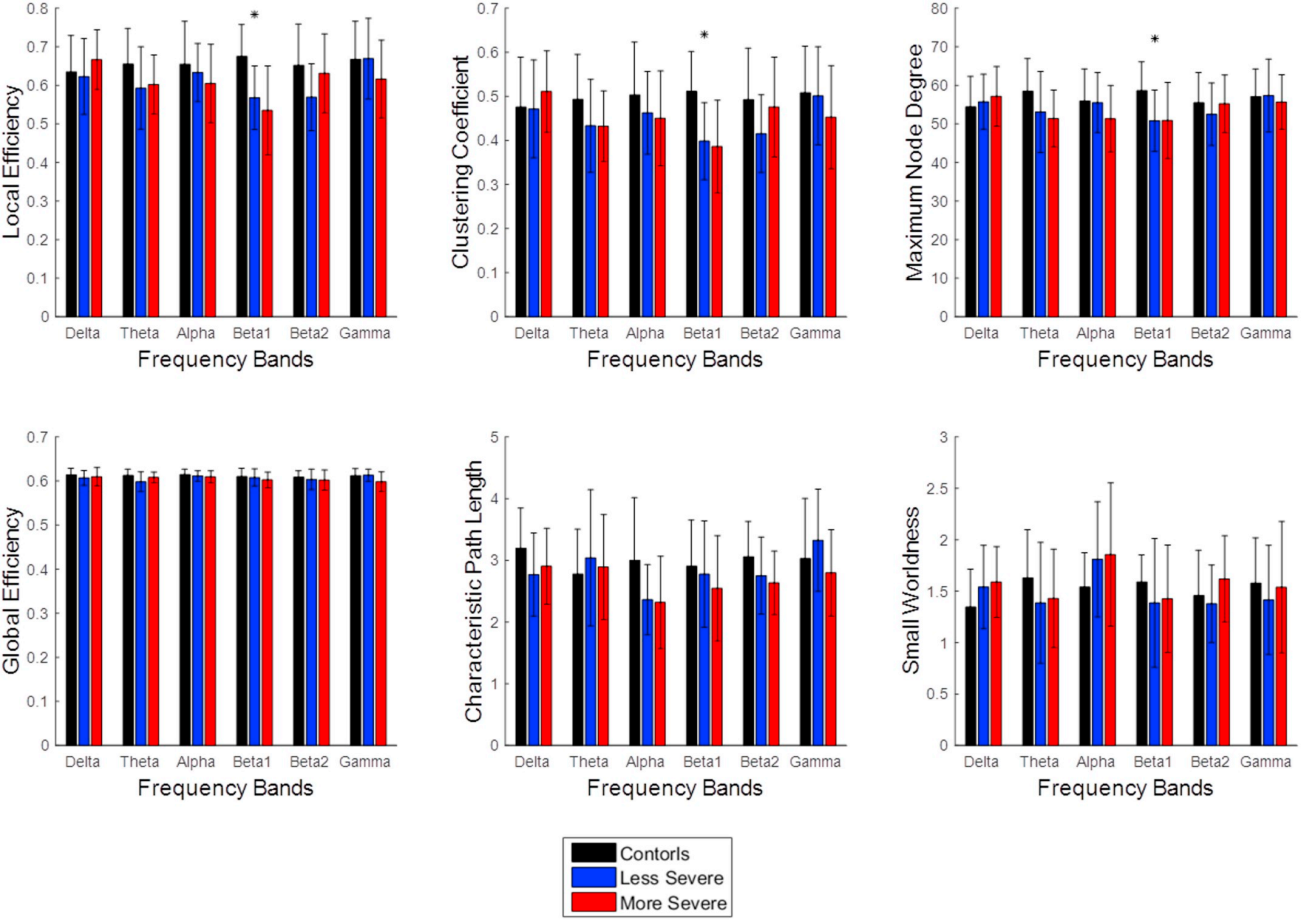
Bar graphs showing graph parameter values across frequencies at the sparsity level S = 0.15. The bars show the average value of the graph parameters and the error bars show the standard deviation. The * indicates a p < .05. The only band to show significant differences in any of the parameters was the beta1 band. The beta1 band was selected to analyze the resting state EEG data.

In order to limit the number of comparisons performed for EEG resting state data, a comparison between the three groups of the six graph theory parameters was performed at S = 0.15, to be consistent with the fMRI resting state analysis. Significant differences were only observed in the beta1 band, shown in Fig. 4. Therefore, EEG resting state analysis was only performed in the beta1 band. The sparsity threshold ranged from 0.10 to 0.30 with a 0.01 increment. The six parameters described in the fMRI resting state analysis were calculated for each subject. A comparison of the groups was done by calculating the mean and standard deviation. The general trends observed in EEG were compared to the trends observed from fMRI.

### 2.8 Statistical analysis

Reporting differences between the clinical parameters for the less severe and more severe sickle pain patient groups were done by using an independent *t*-test. For determining significant differences between groups on the graph theory analysis, functional connectivity, and anatomical analysis an ANOVA test was used followed by multiple comparisons correction using the Tukey method (Tukey's Honestly Significant Difference (HSD), 2010). This process was used to determine significant differences between control, less severe, and more severe groups for the sparsity analysis for fMRI and EEG, the robustness analysis, and determining which frequency band to assess for the resting state EEG analysis. For the induced pain study, this process was also used; however, due to the limited number of participants significant differences should be cautiously considered as an outlier can greatly affect results for a small subject pool. All results with *p* < .05 after corrections are reported as significant. For the linear correlations between the graph parameters and the clinical parameters, the R^2^ and *p*-values were found. Only correlations with p < .05 after false-discovery rate corrections are reported as significant for this study.

To determine regional differences between the groups, tests to look for significant differences between the edges and the nodes were conducted. For edges, the connectivity value for each possible connection was tested for significance by using an ANOVA. Edges with p < .05 after multiple comparison corrections are reported as significant. For nodes, the node degree and clustering coefficient was found for each node in the network. These values were tested for significance using an ANOVA and nodes with p < .05 after multiple comparison corrections are reported as significant.

## 3 Results

### 3.1 Patient statistics

The clinical parameters reported in this study are listed in Table 1. Most of the parameters showed no significant differences between the less severe and more severe patient groups. The number of emergency department visits and hospitalizations, as expected, were significantly different between the groups. However, the pain score recorded the day of the study also showed a significant difference between the groups and the percent fetal hemoglobin showed a trend for difference between the groups. The medications taken by the patient group varied greatly as can be seen in Table 2. Patients were often taking more than one medication. The most common medications taken across the patients included hydroxyurea, folate, oxycodone, and ibuprofen.

### 3.2 Resting state fMRI findings

**Fig. 5:**
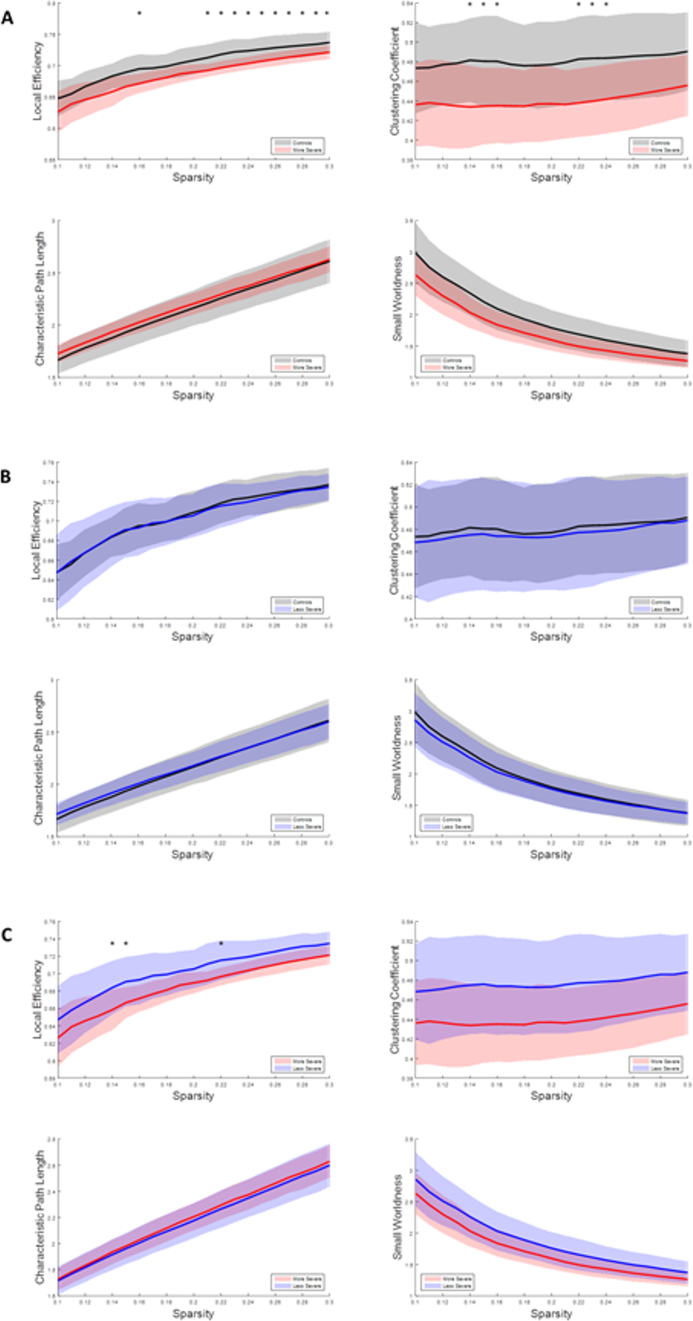
Graphs depicting how graph parameters change with sparsity levels from resting state fMRI. A. The control group and less severe patient group results. B. The control group and more severe patient group results. C. The less severe patient group and more severe patient group results. The lines represent the average values of the group, and the shaded regions represent the standard deviation of the group. The * indicates p < .05.

The sparsity analysis showed that the network properties of the more severe patient group behaved differently compared to the other groups, shown in Fig. 5. Generally, the more severe group had lower local efficiency, and clustering coefficient compared to the control group and the less severe group. There were typically more statistical differences observed between the control and more severe patient groups. No statistically significant differences were observed between the control group and the less severe patient group.

**Fig. 6:**
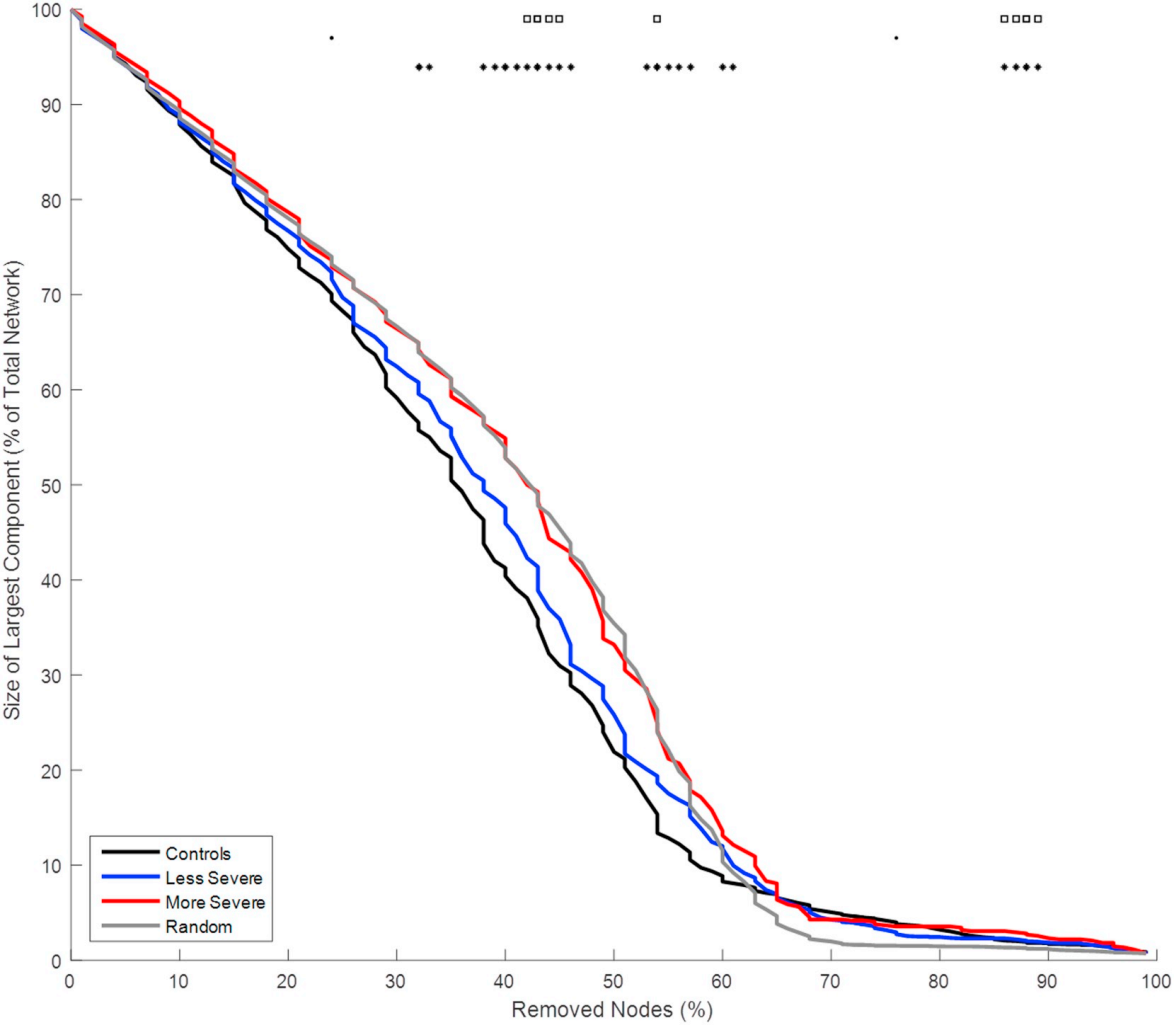
Graph showing robustness analysis results from resting state fMRI. The lines represent the average value of each group. The * represent significant differences (p < .05) between the less severe and more severe groups. The · represent significant differences (p < .05) between the control and more severe groups. The squares represent significant differences (p < .05) between the control and less severe groups.

The robustness analysis showed how each group's network responded to a targeted attack, Fig. 6. The patient groups behaved similarly when a small portion of the nodes had been removed; however, the less severe group shifted toward the control group's response while the more severe group continued to behave similarly to a random network as more nodes were removed. Once a majority of the nodes were removed the control group and less severe group had a smaller main cluster compared to a random network and the more severe group.

**Fig. 7:**
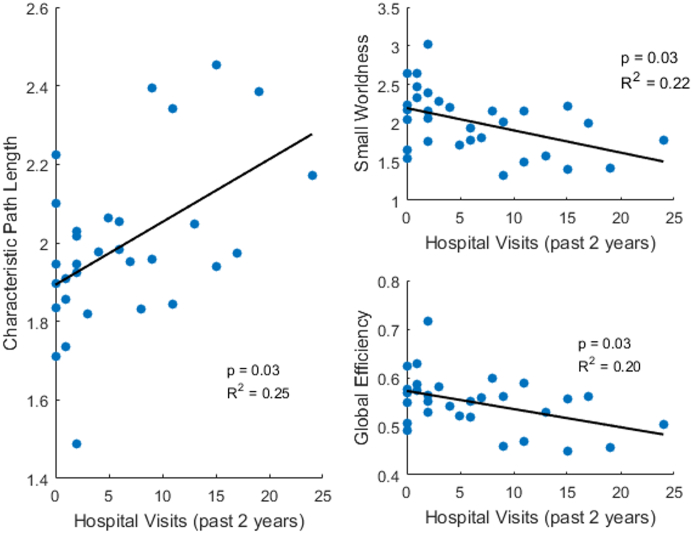
Correlation graph between past hospitalizations and graph parameters from resting state fMRI. The lines represent the linear trend. The significance of the correlation and the R^2^ value are displayed on the charts. Both patient groups are plotted for these graphs.

Three of the graph parameters showed correlations with the number of hospitalizations in the past two years, Fig. 7. The characteristic path length had a positive correlation with the number of hospitalizations. The small world value and the global efficiency had a negative correlation with the number of hospitalizations. The other parameters had no significant correlations.

**Fig. 8:**
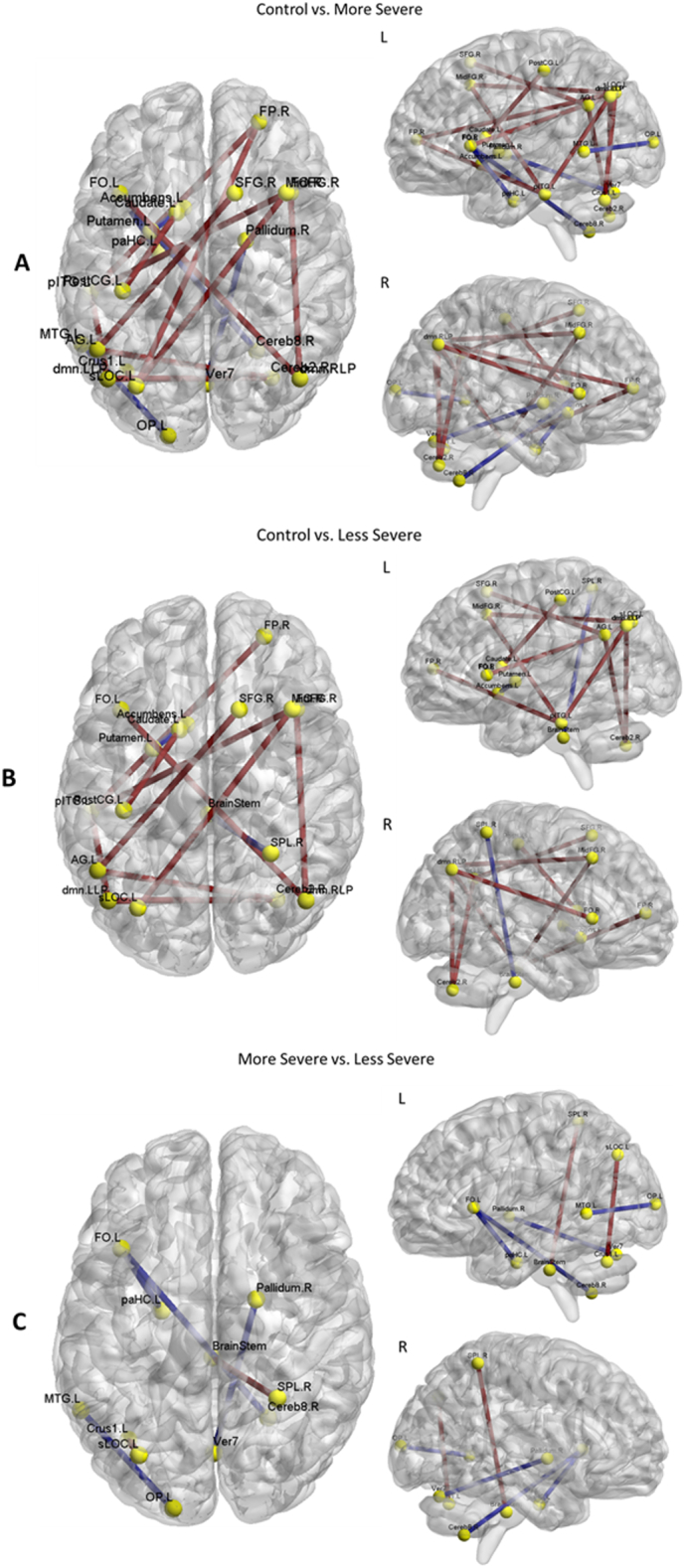
Chart showing significant edges from the resting state fMRI results. A. Significant edges between the control and less severe patient group. Red lines represent where controls have stronger edges and blue lines represent where patients have stronger edges. B. Significant edges between the control group and more severe patient group. Red lines represent where controls have stronger edges and blue lines represent where patients have stronger edges. C. Significant edges between the less severe and more severe patient groups. Red lines represent where less severe patients have stronger edges and blue lines represent where more severe patients have stronger edges. The brain regions for the node labels are listed in Table 4. The images were generated using the BrainNet toolbox.

The control group had more significant interregional edges compared to both patient groups, Fig. 8. The less severe patient group had stronger edges compared to the more severe group, but not to the extent of the controls. The patient groups had some significantly stronger edges compared to controls including the right superior parietal lobe to the brain stem, the left parahippocampal gyrus to the left frontal operculum cortex, the left frontal operculum cortex to the right cerebellum, the left putamen to the left accumbens, and the right pallidum to the vermis 7. The nodes with significantly different node degree and clustering coefficient values are shown in Table 3. The contrast between the controls and more severe patient group showed the greatest number of significantly different nodes. In particular, the clustering coefficient assessment showed that nodes were globally spread throughout the brain. This indicates that the more severe group has less clustering coefficient values, which was also seen in the sparsity analysis.

**Table 3:** Node regions with significant differences between groups.

Node degree		
Controls vs less severe nodes	Controls vs more severe nodes	Less severe vs. more severe nodes
*Right Inferior Frontal Gyrus, pars opercularis*	**Left Frontal Pole**	**Left Frontal Pole**
	**Left Middle Frontal Gyrus**	**Left Middle Frontal Gyrus**
	*Left Parahippocampal Gyrus, anterior division*	**Left Superior Lateral Occipital Cortex**
	*Right Pallidum*	*Left Cerebelum 4 5*
	*Vermis 9*	
	*Vermis 10*	


Clustering coefficient		
Controls vs less severe nodes	Controls vs more severe nodes	Less severe vs. more severe nodes
**Left Superior Lateral Occipital Cortex**	**Right Frontal Pole**	**Right Frontal Pole**
**Right Intracalcarine Cortex**	**Right Middle Frontal Gyrus**	**Right Middle Frontal Gyrus**
**Left Planum Temporale**	**Right Inferior Frontal Gyrus, pars triangularis**	**Right Inferior Frontal Gyrus, pars triangularis**
**Right Supracalcarine Cortex**	**Right Inferior Frontal Gyrus, pars opercularis**	**Left Inferior Temporal Gyrus, temporooccipital part**
*Right Cerebelum 9*	**Right Superior Temporal Gyrus, posterior division**	**Cingulate Gyrus, posterior division**
**Vermis 6**	**Left Middle Temporal Gyrus, anterior division**	**Right Frontal Orbital Cortex**
*Medial Prefrontal Cortex*	**Right Middle Temporal Gyrus, posterior division**	**Right Cerebelum Crus1**
	**Left Superior Lateral Occipital Cortex**	**Medial Prefrontal Cortex**
	**Right Intracalcarine Cortex**	
	**Left Intracalcarine Cortex**	
	**Left Planum Temporale**	
	**Right Supracalcarine Cortex**	

Bold nodes indicate regions where first group listed are higher.

Italicized nodes indicate regions were second group listed are higher.

**Table 4:** Node Labels used for Edge Figures.

Node Label	Brain Region
FP.R	Right Frontal Pole
SFG.R	Right Superior Frontal Gyrus
MidFG.R	Right Middle Frontal Gyrus
MTG.L	Left Middle Temporal Gyrus
pITG.L	Left Posterior Inferior Temporal Gyrus
PostCG.L	Left Postcentral Gyrus
SPL.R	Right Superior Parietal Lobule
AG.L	Left Angular Gyrus
sLOC.L	Left Superior Lateral Occipital Cortex
paHC.L	Left Anterior Parahippocampal Gyrus
FO.R	Right Frontal Operculum Cortex
FO.L	Left Frontal Operculum Cortex
OP.L	Left Occipital Pole
Caudate.L	Left Caudate
Putamen.L	Left Putamen
Pallidum.R	Right Pallidum
Accumbens.L	Left Accumbens
BrainStem	Brain Stem
Crus1.L	Left Cerebellum Crus1
Cereb2.R	Right Cerebellum Crus2
Cereb8.R	Right Cerebellum 8
Ver7	Vermis 7
dmn.LLP	Left Parietal Lobe
dmn.RLP	Right Parietal Lobe

### 3.3 Resting state EEG findings

**Fig. 9:**
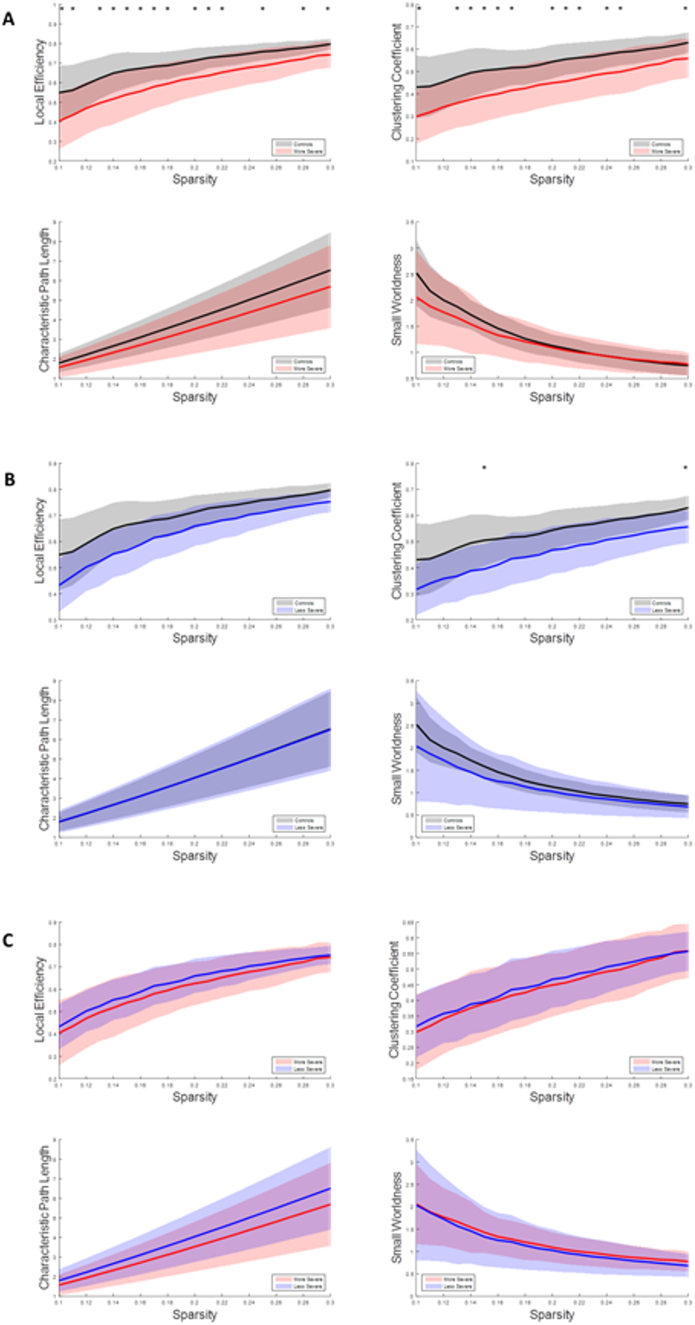
Graphs depicting how graph parameters change with sparsity levels from resting state EEG in the beta1 band. A. The control group and less severe patient group results. B. The control group and more severe patient group results. C. The less severe patient group and more severe patient group results. The lines represent the average values of the group, and the shaded regions represent the standard deviation of the group. The * indicates p < .05.

A sparsity analysis was completed for the beta1 band of the resting state EEG data, Fig. 9. The control group showed significant differences in local efficiency between the more severe patients and significant differences in clustering coefficient between both patient groups, where patient groups had significantly reduced values in these parameters. No significant differences were observed between the less severe and more severe patient group.

### 3.4 Anatomical analysis

**Fig. 10:**
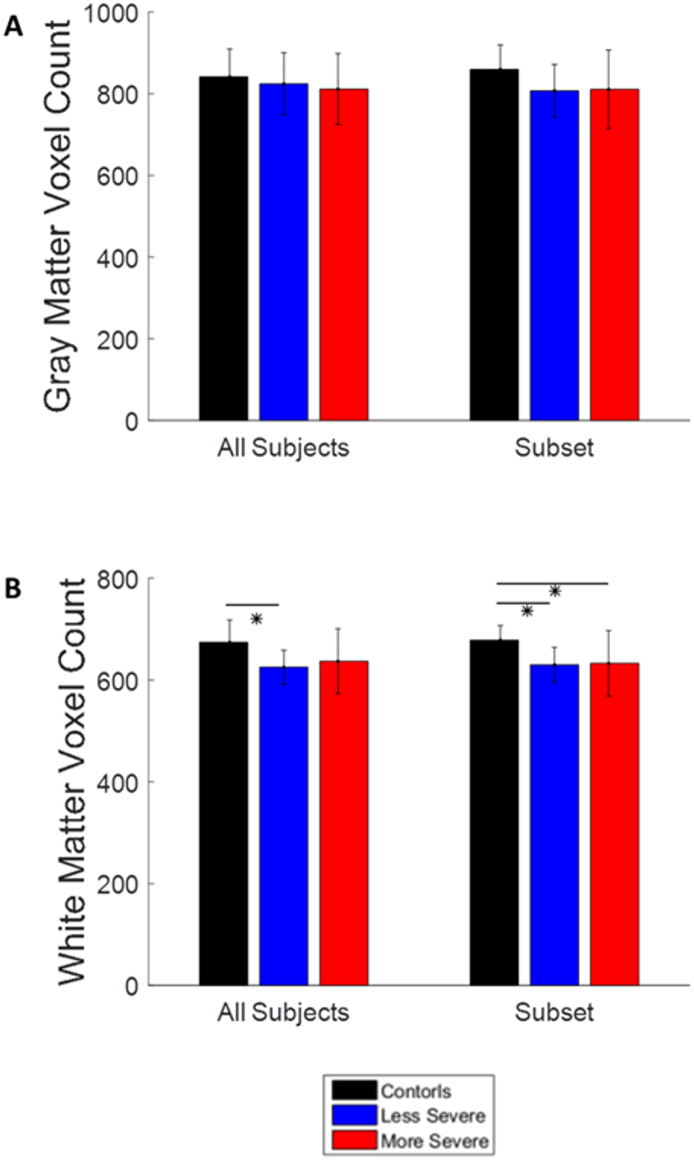
Bar graphs showing the results of the anatomical analysis. A. The average gray matter voxel count of all groups. B. The average white matter voxel count of all groups. The bars show the average value of the functional connectivity and the error bars show the standard deviation. The * indicates a p < .05. The white matter voxel count showed significant differences between the control and patient groups.

The gray matter voxel count did not show any significant differences between any of the groups, Fig. 10A. The subset groups also did not show any significant differences. The control group had significantly more white matter voxels than the less severe patients (*p* = .02) for when all subjects were included, Fig. 10B. The control group had significantly more white matter voxels than the less severe group (*p* = .03) and the more severe group (*p* = .05) for the subset group analysis. This indicates that patients not only have functional neural dynamics, but also anatomical anomalies as well, which may contribute to the disruptions observed in the graph theory analysis.

## 4 Discussion

We have investigated the long-term effects of SCD severity and pain on brain dynamics using non-invasive neuroimaging by means of graph theory analysis. Significant differences were observed between the three groups (control, less severe and more severe disease). Specifically, fMRI showed that the more severe patients had less clustering coefficient and local efficiency values and behaved closer to a random network during a targeted attack. EEG source imaging demonstrated that controls significantly differed from the patient groups in the beta1 band. To our knowledge, this is the first study that uses graph theory to study the global brain network in sickle cell patients utilizing both EEG and fMRI recordings.

The fMRI results found that more severe patients have reduced local efficiency and clustering coefficient compared to controls and less severe patients indicating sickle pain severity impacts global brain dynamics. Altered graph parameter values may reflect why some SCD patients have reduced cognitive abilities compared to controls (Colombatti et al., 2015; Novelli et al., 2015). SCD patients demonstrated altered network behavior in memory-related regions, such as the cerebellum, parahippocampus, and prefrontal cortex observed from the significant edges and nodes analysis, where both patient groups had weaker edges in these regions compared to controls. Additionally, SCD patients have been observed to have declined cognitive performance, which have been linked to imaging through fMRI or gray matter volume analysis (Chen et al., 2017; Colombatti et al., 2016; Mackin et al., 2014; Schatz and Buzan, 2006). Furthermore, another study demonstrated that patients with white matter disease have neuro-architecture with the least similarity to controls and patients without white matter disease (Coloigner et al., 2017). Our anatomical analysis showed that both the less severe and more severe patients had decrease white matter volume compared to controls. This could imply that the decreased white matter may have affected the graph theory parameters.

Small world networks tend to have high clustering and low path lengths, which makes them a robust and efficient network (Achard and Bullmore, 2007; Van den Heuvel and Hulshoff Pol, 2010). The small world values did not show any significant differences between the groups; however, a trend was observed where the more severe patients tended to have decrease small world value compared to the controls. This trend in the more severe patients resulted from decreased clustering across the brain including regions in the frontal cortex, temporal cortex, parietal cortex, occipital cortex, and subcortical regions. The robustness analysis showed how the more severe patient group behaved differently from controls and less severe patients as a result of the loss of small world properties. These more affected patients behaved closer to a random network with low clustering and low path lengths (Van den Heuvel and Hulshoff Pol, 2010). Regions related to the executive control network, emotion-regulation network, and salience network were found to have hyperconnectivity in the patient groups (Damoiseaux et al., 2006; Heine et al., 2012). The salience network, related to pain processing, and the executive control network, related to cognitive processing, have been shown to be altered in sickle cell disease patients (Case et al., 2017; Darbari et al., 2015). Hyperconnectivity may cause disruptions in these resting state networks and this may contribute to the reduced efficiency observed in the patient groups. This suggests that more severe patients, and to a smaller extent the less severe patients, have less organization compared to controls; therefore, patients have a less efficient network.

Several graph parameters were correlated with the number of hospitalizations, an indirect measure of chronic pain history (Ballas et al., 2010; Darbari et al., 2012), during the previous two years. These correlations indicate that long term chronic pain is a factor for abnormal graph parameters. Patients with higher hospitalizations tended to have increased path length and decreased small world values and global efficiency. Patients with migraine showed clustering coefficient and small world value have a positive correlation with the duration of the migraine (Liu et al., 2011; Liu et al., 2012). These results find that graph parameters can reflect factors pertaining to chronic pain. Migraine patients have increased clustering coefficient and small world values compared to controls, the opposite was observed in sickle cell patients. This most likely indicates that the pain mechanisms behind migraine and SCD are different, where migraine patients tend to have increased clustering and stronger edges compared to controls to form a highly interconnected community (Liu et al., 2015). Sickle cell patients have reduced clustering and weaker edges, which most likely leads to reduced connectivity in resting state networks, which has been reported previously (Case et al., 2017).

This study included both fMRI and EEG analysis for resting state data to compare how the two modalities performed. The EEG resting state data indicated significant differences only within the beta1 band. Notably, in Alzheimer's disease, a similar phenomenon was observed where beta band reflected results observed in fMRI, where patients have decreased clustering coefficient and increased path length (Stam et al., 2007). The beta band appears to reflect abnormality in resting state activity in several disorders including Alzheimer's disease, attention deficit disorder, and SCD (Case et al., 2018; Lansbergen et al., 2011; Stam et al., 2005). The EEG resting state results displayed more severe patients had significantly decreased local efficiency and clustering coefficient compared to controls, which is the same result observed in the fMRI results. However, the less severe group also presented significantly reduced clustering coefficient, which was not observed in resting state fMRI, and caused no differences to occur between the patient groups in resting state EEG. Differences between the two modalities were expected as the graph analyses were slightly different. The fMRI graphs had a greater number of nodes, and edge strength was calculated from correlation coefficient rather than the coherence spectrum. Additionally, fMRI has greater spatial resolution while EEG has greater temporal resolution (He and Liu, 2008). However, the major differences observed in fMRI were still seen in EEG, such as reduced clustering. While not all of these differences were significant, such as more severe patients having greater reduction in these parameters compared to less severe patients, graph analysis using both modalities are fairly similar.

Our study has several limitations. We did not impose constraints on how long patients should abstain from taking medication prior to the experiment as it would be unethical to hold treatment for pain for a research study. This introduces a confounder, as the role of pain medication in network alteration cannot be determined. The control group was not ethnically matched to the patient groups; however, the group was ethnically diverse and a large portion of controls were African American. Other complications of SCD, such as ischemia, could cause differences in neural signals. However, we are confident our results reflect neural activity as the graph parameters showed a correlation with the number of hospitalizations and expected trends were found in the induced pain study. The lack of neuropsychology testing is another limitation as we cannot determine if other comorbidities associated with chronic pain, such as depression and anxiety, contribute to the results. Since T2 FLAIR imaging was not performed in this study, we cannot comment on the possible impact of silent cerebral infarctions on the brain network differences observed in our patients. Finally, the settings for fMRI were not the same at the two different locations. This could affect the contrast of the T1 images. An ANOVA test was conducted to determine if the overall functional connectivity was different between the two locations and no significant differences were found. Despite these limitations, we demonstrated significant differences between patients with less severe and more severe disease related pain as well as between patients and controls. We additionally showed correlations between graph parameters and hospitalizations, which reflects pain burden. Our results should be interpreted with caution, but our study provides a foundation for further exploring the long-term impacts of this disease on brain network.

## 5 Conclusions

This study demonstrates that graph theory can be used to assess the overall brain network of SCD patients. It is noteworthy that we found a significant difference between patients and controls, as well as between patients with less severe and more severe SCD, indicating the sensitivity and robustness of this novel non-invasive technique in determining brain function in SCD. Our graph theory results show the impact of long term chronic pain and disease severity on the brain, as the nodes with significant differences in clustering coefficient are not isolated to pain regions or major resting state network nodes. These results further support that SCD affects brain function (Lance et al., 2014; Solh et al., 2016). Neurological symptoms of SCD, such as silent strokes and cerebral infarcts underlying morbidity in children with this disease require attention to diagnose the predisposition to such events before they become life-threatening (Rankine-Mullings et al., 2016). Our study shows that graph theory can be used as a tool to diagnose and test the effectiveness of potential treatments for brain associated disorders in SCD.
